# Advanced surveillance and preparedness to meet a new era of invasive vectors and emerging vector-borne diseases

**DOI:** 10.1371/journal.pntd.0006761

**Published:** 2018-10-25

**Authors:** Rebekah C. Kading, Andrew J. Golnar, Sarah A. Hamer, Gabriel L. Hamer

**Affiliations:** 1 Colorado State University, Department of Microbiology Immunology and Pathology, Fort Collins, Colorado, United States of America; 2 Texas A&M University, College of Agriculture and Life Sciences, Department of Entomology, College Station, Texas, United States of America; 3 Texas A&M University, College of Veterinary Medicine and Biomedical Sciences, College Station, Texas, United States of America; University of Wisconsin Madison, UNITED STATES

## Introduction

**Fig 1:**
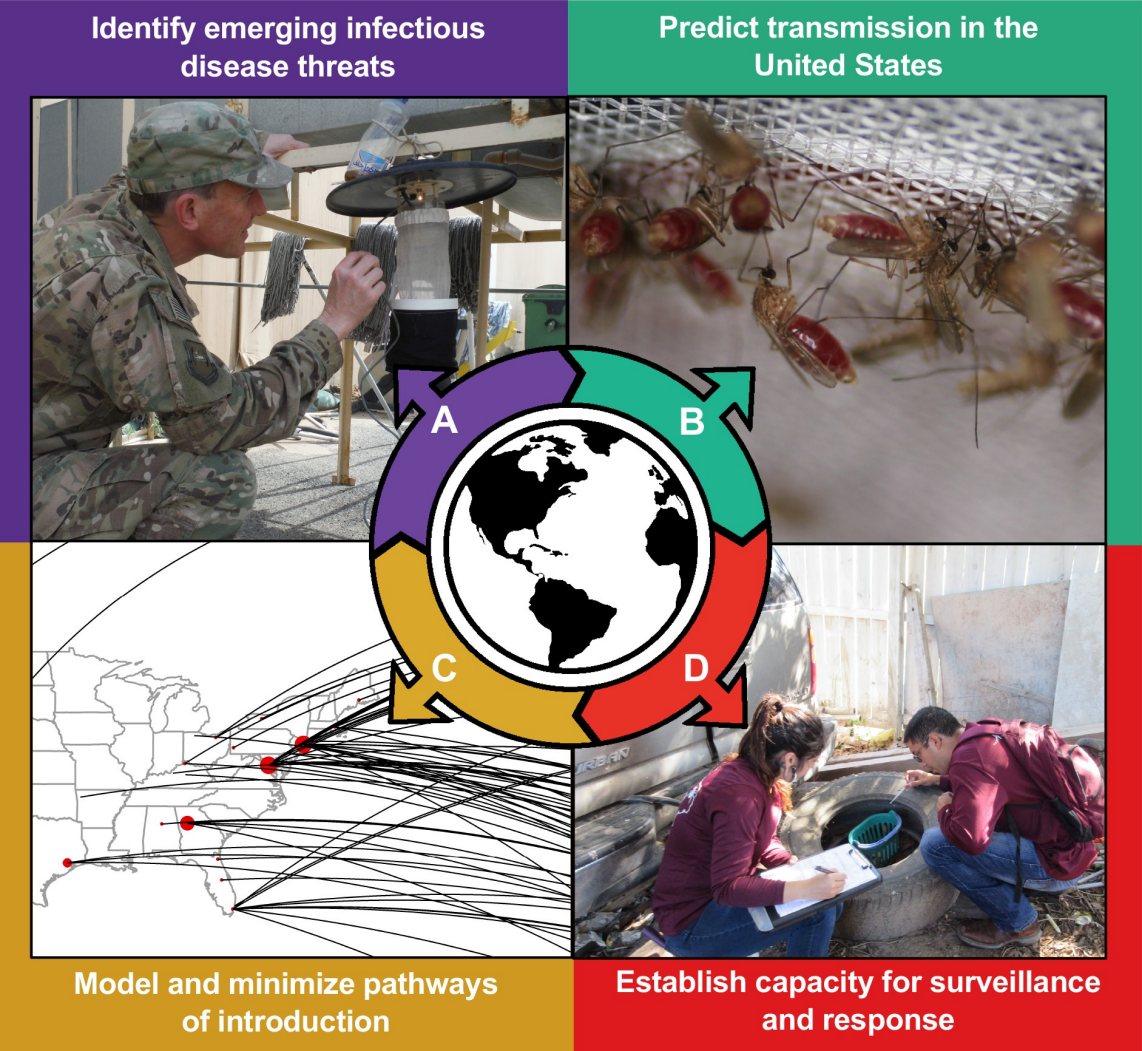
Key elements of a proactive, global surveillance approach. (A) Identification of global vector-borne disease threats, (B) vector and vertebrate competence studies to predict species that would be involved in transmission in the US, (C) modeling pathways of entry [4], and (D) establishment of capacity for surveillance and response. All persons photographed have granted permission to publish their image.

Globalization has increased the rate at which arthropod vectors and associated pathogens emerge and spread. A persistent challenge to the public health agencies in the United States is to identify and control emerging foreign human and animal health threats. This is especially important, given that entomological surveillance capacity in the US has declined in recent years [1]. In this Viewpoint, we promote particular research and capacity-building activities being embraced by scientific and public health communities and funding agencies, which emphasize a local and global mindset for public health preparedness. This global perspective to surveillance looks beyond our borders to what emerging pathogens are circulating and takes a proactive approach to preparing for and reducing these threats. This approach is in contrast to the historical infusions of temporary funding in reaction to an outbreak, which are not sufficient to protect global health security [2,3]. Instead, this framework identifies emerging infectious disease threats, elucidates biological components that would likely support pathogen transmission in a new area, models pathways of pathogen movement, and actively utilizes available data to predict, detect, and mitigate human and animal health threats (Fig 1). Although we focus on vector-borne pathogen invasion and emergence in the US, this concept is globally applicable.

## History of invasive and emerging vector-borne pathogens in the US

Since 1980, many invasive (not native to the US) and emerging (newly recognized or native and expanding in range) vector-borne pathogens have been identified in the US (S1 Table). Some of these invasive pathogens have been extirpated following their detection, some maintain limited autochthonous transmission, and some become endemic (S1 Table). Although invasive public health pathogens tend to experience heightened media coverage (e.g., Zika virus [ZIKV]), the US has also experienced local emergence events of a number of pathogens that have caused significant human morbidity (e.g., *Borrelia* spp. pathogens) (S1 Table). Routine vector surveillance has discovered multiple species of exotic mosquitoes and ticks entering the US, including the Asian tiger mosquito *Aedes (Stegomyia) albopictus* (Skuse) [5], *A*. *(Finlaya) japonicus japonicus* (Theobald) [6], and *A*. *(Finlaya) notoscriptus* (Skuse) [7]. Keirans and Durden [8] reported an alarming 99 species of exotic ticks from 11 genera that were imported into the US during the first half of the 20th century, comprising 4 argasid and 95 ixodid species imported on a diversity of mammal, avian, and reptilian species, trophy hides, commodities, and household goods [8]. Multiple life stages of the Asian longhorned tick (*Haemaphysalis longicornis*)—native to East Asia—were recently found to infest sheep on a farm in New Jersey, with subsequent detections in additional states [9] and unknown disease risk. Migratory birds are also responsible for introducing exotic species of neotropical ticks to the US each spring [10]. As global commerce and connectivity continue to expand, the growing propagule pressure of medically important vectors and pathogens will also grow. Accordingly, resource availability and surveillance strategies must adapt to counteract challenges associated with emerging vector-borne disease pathogens.

## The identification of global emerging vector-borne disease threats

What is the next emerging or invasive pathogen? Surveillance programs should seek to strengthen the global public health network by building capacity for vector and pathogen surveillance, detection, and response in foreign countries as well as on home turf. Multiple US agencies have launched significant global biosecurity initiatives that embody this goal. In particular, the Centers for Disease Control and Prevention (CDC) maintains a significant global presence in pathogen detection, outbreak investigation, and response through the multisectoral efforts of the Global Health Security Agenda to strengthen public health systems and improve pathogen detection and response capacity [11], as well as orchestration of the Field Epidemiology Training Programs (FETP) [12]. The US Agency for International Development (USAID) Emerging Pandemic Threats program works to strengthen capacities in developing countries to predict, identify, prevent, and respond rapidly to infectious diseases before they become significant human health threats [12]. The USAID PREDICT project has focused extensively on improved surveillance for emerging threats in identified hotspots for emerging diseases [12]. The Defense Threat Reduction Agency Cooperative Biological Engagement Program (DTRA-CBEP) works with partner nations to improve biosafety, biosecurity, and disease surveillance for high-consequence and emerging pathogens [13]. Finally, Defense Advanced Research Projects Agency (DARPA)’s Preventing Emerging Pathogenic Threats (PREEMPT) program seeks to predict and prevent cross-species transmission of viral infectious disease from animals and insects to humans [14]. These programs collectively increase our knowledge of vectors and infectious agents in endemic locations prior to their arrival in the US and build global collaborations to combat emerging and invasive diseases that threaten us all. Implementing proactive surveillance programs relies on detailed cost–benefit analyses [15] and prioritization of resources—such as the One Health Zoonotic Disease Prioritization Tool [16]—to communicate strategic goals between public health stakeholders, policy makers, and funding agencies alike.

## Predicting vector-borne disease transmission in the US

What local vector and vertebrate species might contribute to the transmission of invasive or emerging pathogens? Data on vector distributions, habitat associations, seasonal abundance, and blood feeding patterns inform models that assess and predict community risk to pathogen transmission [17–18]. Furthermore, supporting data on the relative competence of different vectors [19] and vertebrate hosts [20] for pathogen transmission is tremendously important for determining the epidemiological significance of field observations and identifying vectors and hosts capable of supporting pathogen invasion. Laboratory competence data and vector–host association data can then be synthesized to predict (based on ecological associations or taxonomic relatedness to the competent species in the endemic area) the likely vectors and vertebrate species that would be involved in the transmission of an introduced pathogen [21–22] or be used to predict spatial and temporal conditions that may promote the transmission and establishment of foreign vector-borne diseases [23]. Information produced by these proactive studies helps develop regional and local agency preparedness, improve response to introduction events, guide the development of countermeasure technologies, and identify critical knowledge gaps.

## Predicting and minimizing the pathways of introduction

How and where will a foreign pathogen arrive? Global movement data can be used to quantitatively predict likely pathways of introduction. Vectors can invade via ship, ground, or air traffic, in some cases while attached to a host; pathogens can be introduced through vectors, contaminated products, or vertebrate hosts (including humans). Investigators have analyzed global movement data of people and animals in the context of pathogen and vector invasions for a number of different pathogen systems [4, 24, 25, 26]; however, these data can often be expensive or difficult to obtain, presenting road blocks to such important analyses. Estimating the likelihood of a pathogen entering by a particular pathway subsequently directs surveillance and prevention measures to intercept these invasions at a port of entry. Examples include screening of humans for symptoms of Ebola virus infection at airports [27], aircraft disinsection [28], mandatory vaccinations, health certifications, quarantine periods for imported animals, and US Customs and Border Protection inspections at ports of entry [29]. The development and execution of these strategies to stop pathogen and vector invasion rely on surveillance data and the accuracy of information used to predict pathways of entry.

## Strengthening surveillance and response for vectors and pathogens

How do we detect and respond to invasive or emerging pathogens? Effective surveillance and response will take an army of professionals to identify invasive species and pathogen introductions, support collection of long-term entomological and virus infection data, and provide local capacity to respond to future outbreak events. The devastating effect that suboptimal surveillance capabilities and public health infrastructure can have on a national and global scale was evident in West African Ebola outbreak in 2014. Without the ability to detect and respond rapidly to emerging pathogens, outbreaks can quickly overwhelm existing capacity [30]. Trained personnel are urgently needed at all levels of government, as well as collaborations with the private sector, nongovernmental agencies, academia, and other institutions with a public health focus. Technical expertise and leadership, effective coordination of government and other partner organizations, and strengthening of pathogen testing and response capabilities have proven vital to epidemic control and are in alignment with the public health system strengthening goals of the Global Health Security Agenda [31]. Beyond technical capabilities, understanding and reducing delays in political action to an outbreak response is also key to mobilizing an effective and rapid response to future public health emergencies [31].

Success of these critical activities relies heavily on maintaining strong and sufficient surveillance capacity in the US. Routine vector surveillance activities for established pathogens also monitor seasonal transmission dynamics, which can guide the deployment of evidence-based intervention measures. The crisis of having alarmingly few trained medical entomologists at federal and local levels to handle these activities became evident in 2015 and 2016 during the height of the ZIKV outbreak response [32]. To address this need, the CDC invested over $50 million to create five CDC Regional Centers of Excellence to train the next generation of public health entomologists and to fund the American Mosquito Control Association to “Train the Trainer.” With this emphasis on training a workforce of medical entomologists, it will be critical to ensure that there are positions available for these newly trained professionals to fill and that these positions are maintained. This is particularly challenging given the variability in size, operational capacity, and public-sector–governing vector-abatement activities in the US [33]. Aligning key program activities with operational needs is paramount. One way to address this concern is to make sure the size of the organized vector control for a community is reflective of the local citizen willingness to pay for these programs.

Additionally, pathogen detection strategies need testing algorithms that permit the identification of unknown agents so novel pathogens will not be missed. The traditional approach of virus isolation remains valuable today for this purpose, although it lacks the high-throughput capacity of PCR-based screening and has biosafety considerations. Next-generation sequencing (NGS) offers a powerful technology towards the identification of unknowns and the simultaneous detection of multiple circulating pathogens [34]. Still, NGS is cost-prohibitive to many labs for routine use and requires expertise in bioinformatics and extensive computing capabilities to manage data. These capabilities are improving all the time, such as with field-deployable devices such as the MinION (Oxford Nanopore Technologies), which makes this technology much more accessible. Already, insights from genomic epidemiology studies have contributed new knowledge to our understanding of the epidemiology of ZIKV introduction and transmission in Florida in 2016. Grubaugh and colleagues [35] sequenced ZIKV genomes from infected patients and *A*. *aegypti* mosquitoes and revealed that at least 4—but as many as 40—introductions contributed to the outbreak in Florida and that local transmission is likely to have started several months before its initial detection. Multiplex assays that screen for multiple high-priority pathogens at once, such as screening pools of *A*. *aegypti* mosquitoes simultaneously for ZIKV, chikungunya virus (CHIKV), and dengue virus (DENV), can also make high-throughput processing more efficient [36–37].

## Conclusion

Vector-borne disease agents will continue to emerge globally and invade new places; it is not a matter of “if” but “when.” Through multiple current initiatives, international consortia are working to strengthen global health security by building partnerships and capacity to control emerging infectious diseases before they become pandemics. In addition to building pathogen detection and response capacity, proactive research agendas should assess what invasive pathogens threaten the US and by what pathways these agents would arrive to inform local and national surveillance and response infrastructure. Having diagnostic tools and targeted surveillance strategies in place for rapid roll-out is critically important once a high-priority pathogen is identified. As such, support for global health security needs to be unwavering; the ephemeral infusion of funds that are reactionary to threats are not sufficient to maintain necessary infrastructure required to identify and mitigate emerging and reemerging disease threats.

## Supporting information

S1 Table First detections of vector-borne human pathogens emerging in the US since 1980. (DOCX)
