# Supplementary material for: Advanced surveillance and preparedness to meet a new era of invasive vectors and emerging vector-borne diseases
Source: PLoS Negl Trop Dis. 2018 Oct 25;12(10):e0006761. doi: 10.1371/journal.pntd.0006761 (PMC6201877; doi:10.1371/journal.pntd.0006761)
Supplement: S1 Table — (DOCX) [file pntd.0006761.s001.docx]

(Supplementary Material)

**S1 Table. First detections of vector-borne human pathogens emerging in the United States since 1980.**

Supplemental References

38. Burgdorfer W, Barbour AG, Hayes SF, Benach JL, Grunwaldt E, Davis JP. Lyme disease-a tick-borne spirochetosis? Science 1982:216: 1317-1319.

39. Anderson BE, Dawson JE, Jones DC, Wilson KH. *Ehrlichia chaffeensis*, new species associated with human ehrlichiosis. J Clin Microbiol. 1991:29: 2838-2842.

40. Anderson BE, Greene CE, Jones DC, Dawson JE. *Ehrlichia ewingii* sp. nov., the etiologic agent of canine granulocytic ehrlichiosis. Int J Syst Bacteriol. 1992;42: 299-302.

41. Chen S-M, Dumler JS, Bakken JS, Walker DH. Identification of a granulocytotropic *Ehrlichia* species as the etiologic agent of human disease. J Clin Microbiol. 1994:32: 589–595.

42. Margos G, Fedorova N, Kleinjan JE, Hartberger C, Schwan TG, Sing A, Fingerle V. *Borrelia lanei* sp. nov. extends the diversity of *Borrelia* species in California. Int J Syst Evol Microbiol. 2017: 67:3872-3876.

43. Lanciotti RS, Roehrig JT, Deubel V, Smith J, Parker M, Steele K, et al. Origin of the West Nile virus responsible for an outbreak of encephalitis in the northeastern United States. Science. 1999:286:2333-2337.

44. Scoles GA, Papero M, Beati L, Fish D. A relapsing fever group spirochete transmitted by *Ixodes scapularis* ticks. Vector Borne Zoonotic Dis. 2001:1: 21–34.

45. Morbidity Mortality Weekly Report MMWR. Dengue hemorrhagic fever on the US- Mexican border 2005. MMWR 2007:56:785-789.

46. ArboNET. CDC. 2018. Available from: <https://wwwn.cdc.gov/arbonet/maps/ADB_Diseases_Map/index.html>.

47. McMullan LK, Folk SM, Kelly AJ, MacNeil A, Goldsmith CS, Metcalfe MG, et al. A new phlebovirus associated with severe febrile illness in Missouri. N Engl J Med. 2012:367: 834–841.

48. Pritt BS, Respicio-Kingry LB, Sloan LM, Schriefer ME, Replogle AJ, Bjork J, et al. *Borrelia mayonii* sp. nov., a member of the *Borrelia burgdorferi* sensu lato complex, detected in patients and ticks in the upper midwestern United States. Int J Syst Evol Microbiol. 2016:66:4878-4880.

49. Kendrick K, Stanek D, Blackmore C. Notes from the field: Transmission of chikungunya virus in the continental United States--Florida, 2014. MMWR Morb Mortal Wkly Rep. 2014:63:1137.

50. Kosoy O, Lambert AJ, Hawkinson DJ, Pastula DM, Goldsmith CS, Hunt DC, et al. Novel thogotovirus associated with febrile illness and death, United States, 2014. Emerg Infect Dis. 2015:21(5):760-764.

51. Centers for Disease Control (CDC). Zika virus. 2016. Available from: <http://www.cdc.gov/zika/index.html>.
